# Global Transcriptomic Analysis of the Canine *corpus luteum* (CL) During the First Half of Diestrus and Changes Induced by *in vivo* Inhibition of Prostaglandin Synthase 2 (PTGS2/COX2)

**DOI:** 10.3389/fendo.2019.00715

**Published:** 2019-11-13

**Authors:** Miguel Tavares Pereira, Felix R. Graubner, Hubert Rehrauer, Tomasz Janowski, Bernd Hoffmann, Alois Boos, Mariusz P. Kowalewski

**Affiliations:** ^1^Vetsuisse Faculty, Institute of Veterinary Anatomy, University of Zurich, Zurich, Switzerland; ^2^Functional Genomics Center Zurich (FGCZ) ETH/UZH, Zurich, Switzerland; ^3^Department of Animal Reproduction, University of Warmia and Mazury, Olsztyn, Poland; ^4^Clinic for Obstetrics, Gynaecology and Andrology, Faculty of Veterinary Medicine, Justus Liebig University, Giessen, Germany

**Keywords:** canine (dog), *corpus luteum*, prostaglandins, transcriptome (RNA-seq), diestrus

## Abstract

The canine luteal phase exhibits several peculiarities compared with other species. In early diestrus, the *corpus luteum* (CL) is, at least in part, independent of gonadotropins, and prostaglandins (PGs) appear to be among its main regulators. This was also observed with the inhibition *in vivo* of COX2, when also transcriptional capacity, vascularization and immune-related factors were affected. Here, we aimed to further investigate the potential effects of PGs withdrawal on the CL transcriptome by performing deep RNA sequencing (RNA-Seq). Samples from a previous *in vivo* study were used; bitches were treated for 5, 10, 20, or 30 days after ovulation with firocoxib (Previcox®), a PTGS2/COX2 inhibitor, or a placebo. Analysis of results was performed with SUSHI (framework from FGCZ) and with pathways and functional networks analyzers. Time-dependent effects were also investigated and used for quality control. More highly represented differentially expressed genes (DEGs, *P* < 0.01, FDR < 0.1) in the early CL (days 5 and 10) referred to proliferation and immune system, while in the mature CL (days 20 and 30) they were related with steroidogenesis. The absence of genes concomitantly affected by the treatment at all time-points suggested stage-dependency in the observed effects. Little effect was observed on days 5 and 10. Day 20 had the highest number of DEGs (*n* = 1,741), related with increased immune response. On day 30, DEGs found (*n* = 552) referred to decreased steroidogenesis and vascularization. Our results suggest the presence of strong compensatory effects in the early CL and multidirectional effects toward gonadotropin-dependency of the CL after COX2 inhibition.

## Introduction

The *corpus luteum* (CL) has a central role in pregnancy through its production of progesterone (P4). In dogs, it plays an even more prominent role because it regulates the entire diestric phase. Indeed, the absence of placental steroidogenic activity in this species makes the CL the sole source of pregnancy-supporting P4 (1, 2). Following exceptionally strong preovulatory luteinization (3), the CL continues to develop following ovulation. As in other species, this is supported by the formation of a dense vascular network (4, 5). Serum P4 levels vary greatly among animals and peak between days 15 and 30 after ovulation. Shortly thereafter, luteal function starts to diminish, accompanied by decreasing levels of P4 and signs of cellular degeneration (6–8). The function of the CL is actively terminated in pregnant bitches shortly before parturition (around day 60 after ovulation) during prepartum luteolysis. This is associated with increased circulating prostaglandin (PG)F2α, apparently produced by the fetal placenta (1, 9–12). Interestingly, no such active mechanism is observed in the absence of pregnancy (12, 13). There is no uterine luteolysin in non-pregnant dogs and hysterectomy does not affect CL function (12, 13). Consequently, non-pregnant bitches present a physiological pseudopregnancy, with circulating P4 levels similar to those in pregnant animals (14, 15). It appears thus, that in lacking an active luteolytic principle, the CL life span of non-pregnant dogs is regulated by some intrinsic regulatory mechanisms. At the regulatory level, the canine CL also presents some species-specific peculiarities compared with other domestic animals. Both LH and prolactin (PRL) have luteotropic roles in the canine CL (16–18). However, PRL appears to be the predominant factor and appears to be required for CL maintenance starting around day 25 after ovulation (19, 20). It is noteworthy that ablation of the hypophysis had less effect on CL function in the first 2–4 weeks after ovulation (17). Consequently, the canine CL presents a transitory independence on gonadotropins in its earlier stages, progressing to a gonadotropin-dependent stage at mid-diestrus, with PRL then acting as the predominant luteotropic factor (17, 18, 20).

The initial observation of increased expression of COX2/PTGS2 and PGE2 synthase (PTGES) in early CL stages suggested PGs as possible important regulators of this organ in the dog (21, 22). Further investigations showed direct effects of PGE2 in early canine luteal cells, in which it could increase steroidogenic acute regulatory (STAR) protein expression and P4 production (23). Following these clues, the effects of PGs on CL function were explored *in vivo* (3, 24). For this, firocoxib (Previcox, Merial Ltd.), a specific inhibitor of COX2, was used to treat bitches from the day of ovulation up to 30 days later, with the aim of causing functional withdrawal of PGs (3, 24). In fact, the expression of PTGES and intra-CL levels of PGE2 and PGF2α were significantly decreased by this treatment (3). It also affected the steroidogenic capacity of the CL (decreased expression of 3βHSD and STAR), and suppressed the levels of circulating P4 (3, 24). Furthermore, the decreased nuclear size of luteal cells induced by this treatment was related to their decreased transcriptional capacity (24). These observations showed a causality between COX2 and the PTGES-dependent synthesis of PGE2 in the CL and established PGs as important modulators of CL function (3, 24).

It appears, however, that PGs might have broader effects on the regulation of CL function in addition to steroidogenesis. COX2 inhibition decreased the expression of the PRL receptor (PRLR) while, in parallel, PGE2 could increase the expression of PRLR in isolated canine luteal cells (3). This suggested an indirect role of PGE2, through the enhancement of PRLR expression, to support the luteotropic function of PRL. Based on this, and on the fact that PGE2 could also increase the expression of endothelin receptor B (ETB; important vasodilator) (25), the effects of PGs withdrawal on luteal vascular and immune factors were further investigated (26). The stabilization of blood vessels was negatively affected by the treatment, while the expression of different pro-inflammatory factors was increased (26). Concomitantly, the presence of strong compensatory effects was implied.

Considering the peculiarities of the canine luteal phase, the present study aimed to investigate additional potential effects of withdrawal of PGs on CL physiology. For this, we used samples from a previously reported *in vivo* study (3, 24, 26), and performed a deep RNA sequencing (RNA-Seq) by applying Next Generation Sequencing (NGS) technology. With this approach, we expected to provide deeper understanding of the importance of PGs at different stages of CL development: its early gonadotropin-independence, then its transition to dependence on hypophyseal hormones, and finally the dependence of the mature CL mainly on PRL. Time-dependent changes in the CL transcriptome associated with its development were also investigated in control samples.

## Materials and Methods

### Tissue Samples

This is a follow-up study utilizing tissue material collected in preceding *in vivo* studies (3, 24, 26). Animal experiments were approved by the responsible ethics committee (permit 54/2008) of the University of Warmia and Mazury in Olsztyn, Poland. Briefly, mixed-breed bitches of different ages (2–7 years old) were monitored by vaginal cytology and P4 measurements for the onset of spontaneous estrus. The day when circulating P4 levels exceeded 5 ng/ml was considered the day of ovulation (day 0). Animals were then randomly assigned to four control and four treated groups, receiving orally and daily either a placebo or 10 mg/kg of firocoxib (Previcox, Merial Ltd), respectively. Treatment was maintained for 5, 10, 20 or 30 days after ovulation. Ovaries were collected by ovariohysterectomy on the last day of treatment. *Corpora lutea* were dissected from surrounding ovarian tissue and preserved in RNAlater at –80°C until further use, as described before (23).

### RNA Isolation and Purification

TRIzol reagent (Invitrogen, Carlsbad, CA, USA) was used to isolate total RNA, following the manufacturer's instructions. A primary evaluation of RNA purity and concentration was performed with a NanoDrop 2000C spectrophotometer (ThermoFisher Scientific AG, Reinach, Switzerland). Further purification of RNA was performed with the RNeasy Mini Kit (Qiagen GmbH, Hilden, Germany), using the RNA Cleanup protocol provided. RNA integrity was assessed with the Agilent 2200 TapeStation System. RNA integrity numbers (RIN) ranged from 8.0 to 9.8.

### RNA-Sequencing and Data Evaluation

#### Next Generation Sequencing (NGS, RNA-Seq)

Sequencing of RNA with NGS was performed to obtain a quantitative evaluation of gene expression. A total of 32 samples available for this follow-up study were allotted to the following groups: *n* = 5 for day 5 control, *n* = 4 for day 5 treated, *n* = 4 for either day 10 control and day 10 treated, *n* = 3 for either day 20 control and day 20 treated, *n* = 4 for day 30 control and *n* = 5 for day 30 treated. RNA-Seq was performed on *n* = 4 animals/group (control and treated) from days 5, 10, and 30, and *n* = 3 animals/group from day 20 after ovulation. Library preparation, cluster generation and sequencing were performed as previously described (27). All samples were processed at the same time to avoid possible batch effects. In brief, the Qubit (1.0) Fluorometer (Life Technologies, Carlsbad, CA, USA) and Bioanalyzer 2100 (Agilent, Waldbronn, Germany) were used to evaluate the quantity and quality of isolated RNA. To be processed further, samples needed to have a 260/280 nm ratio between 1.8 and 2.1 and a 28S/18S ratio within 1.5–2.0. In the succeeding steps, the TruSeq RNA Sample Prep Kit v2 (Illumina, Inc., San Diego, CA, USA) was used. Total RNA samples (100–1,000 ng) were enriched by poly-A selection and reverse-transcribed to obtain double-stranded cDNA. Additionally, fragmentation, end-repair and polyadenylation steps were performed on cDNA samples before ligation of TruSeq adapters containing the index for multiplexing. PCR was used for selective enrichment of fragments containing TruSeq adapters on both ends, and the quality and quantity of the enriched libraries obtained were assessed with the Qubit (1.0) fluorometer and Caliper GX LabChip GX (Caliper Life Sciences Inc., Hopkinton, MA, USA). Finally, libraries were normalized to 10 nM in Tris-Cl 10 mM with Tween 20.

Cluster generation was performed with 2 nM of pooled normalized libraries on the cBOT System with the TruSeq PE Cluster Kit v4-cBot-HS or TruSeq SR Cluster Kit v4-cBot-HS (Illumina, Inc). Sequencing was performed with the Illumina HiSeq4000 with single end 125 bp using the TruSeq SBS Kit v4-HS (Illumina, Inc.). The raw data (.fastq files) obtained were used for downstream analysis. Additionally, they were also deposited in NCBI's Gene Expression Omnibus and are accessible through GEO Series accession number GSE130369.

#### Data Analysis

The initial analysis of data was performed with the framework SUSHI (28, 29), developed by the Functional Genomics Center of Zurich (FGCZ ETH/UZH, Zurich, Switzerland). Spliced Transcripts Alignment to a Reference (STAR) software was used to align the RNA-Seq dataset (30) to a reference canine genome, the Ensembl genome build CanFam3.1 (http://www.ensembl.org/Canis_familiaris/Info/Index). Gene expression values were obtained with the function *featureCounts* from the R package Rsubread (31). A minimum average of 10 reads in at least one group of replicates was required to consider a gene as detected. After reads counting, initial quality control and explorative analysis were performed with the SUSHI framework.

For differential expression analysis different contrasts (pairwise comparisons) were defined and gene differential expression for each contrast was assessed with the generalized linear model approach from the Bioconductor package DESeq2 (32). This was performed as previously described (27), using the Wald test to assess the significance of differential expression. Next, correction of multiple testing was obtained with the Benjamini-Hochberg algorithm that computes False Discovery Rate (FDR, adjusted *P*-value). Finally, thresholds of *P*-value < 0.01 and adjusted *P*-value < 0.1 (i.e., FDR < 10%) were applied. The complete differentially expressed genes (DEGs) lists obtained for each contrast were used for downstream analyses and are provided in Supplemental Table 1 (for time-dependent effects) and Supplemental Table 2 (for treatment-induced effects).

Functional characterization of DEGs for each contrast was performed by identifying different functional terms, resorting to available bioinformatics resources. The identification of over-represented functional categories (i.e., gene ontologies) and their enrichment scores were calculated using the web-based Panther software [http://pantherdb.org, (33)]. This analysis was further supported and complemented with the web-based software Enrichr [http://amp.pharm.mssm.edu/Enrichr/, (34)]. The identification and visualization of enriched functional biological networks was obtained with the application ClueGO v2.5.1 (35) for the software platform Cytoscape v3.6 (36). The software Ingenuity Pathway Analysis (IPA, Qiagen, Redwood City, CA, USA) was used to predict the most significantly affected canonical pathways and identify possibly involved upstream regulators. Lists of up to 10 representative genes involved with particular functional terms (gene ontologies, functional networks, canonical pathways and upstream regulators) from each contrast are presented in Supplemental Table 3. In addition, Venn diagram was generated to identify DEGs concomitantly affected by treatment in different contrasts. For this analysis, the thresholds were defined for *P*-value < 0.01 and fold-change for up- (log2Ratio ≥ 1) and down-regulation (log2Ratio < −1). Full lists of DEGs used as input for Venn diagram and genes present on each intersection are provided in Supplemental Table 4.

### Expression of Selected Target Genes by Semi-quantitative Real-Time TaqMan qPCR

Further validation of the RNA-Seq data obtained and analysis of specific functional pathways suggested by our analysis were performed through evaluation of the mRNA expression of 20 selected candidate target genes, by semi-quantitative real-time (TaqMan) qPCR. All available samples were used for qPCR experiments (*n* = 32). A complete list of primers used, TaqMan probes and pre-designed (i.e., commercially-available) TaqMan systems is presented in Table 1. Self-designed primers and 6-carboxyfluorescein (6-FAM) and 6-carboxytetramethylrhodamine (TAMRA) labeled probes were designed and ordered from Microsynth AG (Balgach, Switzerland). The design of *IDO1* primers and probes was based on published canine CDS sequences while for *SULT1E1* molecular cloning of the canine sequence was required and this was performed by a subcloning approach using the pGEM-T vector, as described previously (22, 37, 38) (sequence submitted to GenBank under accession number MK728829). Commercially-available systems were obtained from Applied Biosystems. The efficiency of PCR reactions was evaluated to ensure approximately 100% (39).

**Table 1:** List of gene symbols, corresponding gene names and TaqMan systems used for real time qPCR.

**Gene**	**Name**	**Accession number**	**Primer sequence**		**Product length (bp)**
*IDO1*	Idoleamine 2,3-dioxygenase 1	XM_532793.5	Forward	5′-TGA TGG CCT TAG TGG ACA CAA G-3′	116
			Reverse	5′-TCT GTG GCA AGA CCT TTC GA-3′	
			TaqMan probe	5′-CAG CGC CTT GCA CGT CTG GC-3′	
*SULT1E1*	Sulfotransferase family 1E	MK728829	Forward	5′-AAC AGA TGG CAT CTC CTA GAG TAG TG-3′	100
	member 1		Reverse	5′-CGG CAA AGA TAG ATC ACC TTA CAG T-3′	
			TaqMan probe	5′-CCA TCT GCC AGT TGA ACT TCT TCC AGC C-3′	
*TBXAS1*	Thromboxane A synthase 1	XM_005629559.1	Applied Biosystems, prod. no. Cf01022701_m1		105
*PTGDS*	Prostaglandin D synthase	NM_001003131.1	Applied Biosystems, prod. no. Cf02622002_m1		85
*TGFβ1*	Transforming growth factor beta 1	NM_001003309.1	Applied Biosystems, prod. no. Cf02623324_m1		83
*TGFβR1*	Transforming growth factor beta receptor 1	XM_014117881.1	Applied Biosystems, prod. no. Cf02687913_m1		110
*ICAM1*	Intercellular adhesion molecule 1	NM_001003291.1	Applied Biosystems, prod. no. Cf02690470_u1		124
*NODAL*	Nodal growth differentiation factor	XM_546146.3	Applied Biosystems, prod. no. Cf02711306_u1		149
*FAS*	Fas cell surface death receptor	XM_005636650.1	Applied Biosystems, prod. no. Cf02651136_m1		118
*FASLG*	Fas ligand	NM_001287153.1	Applied Biosystems, prod. no. Cf02625215_s1		89
*NFκB1*	Nuclear factor kappa B subunit 1	NM_001003344.1	Applied Biosystems, prod. no. Cf02689968_m1		119
*NFκBIa*	NFKB inhibitor alpha	XM_537413.5	Applied Biosystems, prod. no. Cf02741714_m1		129
*PDGF B*	Platelet derived growth factor subunit B	NM_001003383.1	Applied Biosystems, prod. no. Cf02626637_m1		109
*FGF1*	Fibroblast growth factor 1	XM_014108102.1	Applied Biosystems, prod. no. Cf02716346_g1		77
*FGF2*	Fibroblast growth factor 2	XM_003432481.3	Applied Biosystems, prod. no. Cf03460065_g1		147
*THBS1*	Thrombospondin 1	XM_544610.5	Applied Biosystems, prod. no. Cf02701399_m1		88
*PPARγ*	Peroxisome proliferator activated receptor gamma	NM_001024632.2	Applied Biosystems, prod. no. Cf02625640_m1		92
*EGLN1*	PHD2/egl-9 family hypoxia inducible factor 1	XM_546089.4	Applied Biosystems, prod. no. Cf02713521_m1		115
*NR4A1*	Nuclear receptor subfamily 4 group A member 1	NM_001003227.1	Applied Biosystems, prod. no. Cf02719047_s1		113
*HSD17B7*	Hydroxysteroid 17β dehydrogenase 7	XM_014111264.1	Applied Biosystems, prod. no. Cf02657821_m1		82
*PTK2*	Protein tirosine kinase 2	XM_005627993.2	Applied Biosystems, prod. no. Cf02684608_m1		104
*EIF4H*	Eukaryotic translation initiation factor 4H	XM_014114129.1	Applied Biosystems, prod. no. Cf02713640_m1		136
*KDM4*	Lysine (K)-specific demethylase 4A	XM_005629106.2	Applied Biosystems, prod. no. Cf02708629_m1		96

Elimination of genomic DNA contamination, reverse transcription (RT) and semi-quantitative real-time TaqMan qPCR (RT-qPCR) were performed following the manufacturers' and our previously published protocols (22, 39). Briefly, total RNA samples were treated with the RQ1 RNase-free DNase kit (Promega, Dübendorf, Switzerland) to eliminate possible genomic DNA contamination. Random hexamers were used as primers in the subsequent RT reactions, employing reagents obtained from Applied Biosystems. For RT-qPCR, all samples were run in duplicates in 96-well optical plates, using autoclaved water and RNA not subjected to RT (minus-RT control) instead of cDNA as negative controls. Reactions were run in an automated fluorometer ABI PRISM 7500 Sequence Detection System (Applied Biosystems) and were set as follows: initial denaturation for 10 min at 95°C followed by 40 cycles of 15 s at 95°C and 1 min at 60°C each. Relative quantification was done by applying the comparative Ct method (ΔΔCt). RT-qPCR data were normalized with three reference genes: PTK2, EIF4H, and KDM4. Based on the RNA-Seq data collected herein, these genes were found to be stably expressed in all samples examined using the online tool RefFinder and NormFinder software (40, 41). Since RT-qPCR data were unevenly distributed, logarithmic transformation was performed and results are presented as geometric means (Xg) ± geometric standard deviation (SD). The evaluation of treatment-induced effects was performed with a two-tailed Student's *t*-test, comparing the treatment group with the control group at each time point (day). Additionally, time-related changes in the expression of target genes were evaluated in control animals using the Kruskal-Wallis test (non-parametric ANOVA) followed by Dunn's test. All statistical tests were performed with GraphPad 3 (GraphPad Software Inc., San Diego, CA, USA) and values of *P* < 0.05 were considered statistically significant.

## Results

### Initial Evaluation of Sequencing Results

**Figure 1:**
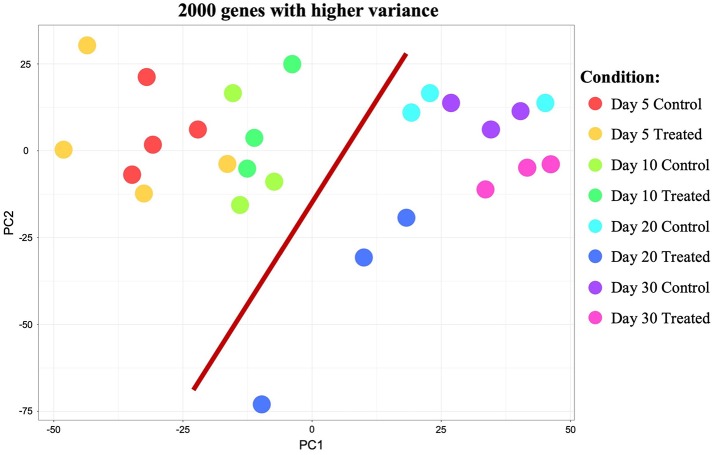
Principal component analysis (PCA) plot of all samples showing the 2,000 genes that presented higher variance. Distribution of samples appears to be mainly based on the effect of time (highlighted by red bar separating samples from days 5 and 10 from samples from days 20 and 30). Samples distribution and scattering also suggest stronger effects of treatment on day 20 than on the other time points studied.

Initial exploratory analysis of the sequencing data obtained was performed with the CountQC app provided in the SUSHI framework. This function allowed assessment of homogeneity and correlations between samples, as well as clustering of high variance features among all samples submitted for RNA-Seq. The analysis of samples correlation matrix (Supplemental Image 1A) indicated the presence of some variation among all sequenced samples. Based on the analysis of this matrix and gene expression scatter plots (not shown), samples 10/1 and 30/1 controls, and 10/2 and 30/1 treated with Previcox, were considered outliers and removed from the dataset for further analysis. This allowed a more homogeneous correlation between samples within each group (Supplemental Image 1B). Further observation of the correlation matrix indicated a higher homogeneity among samples of each control group compared with the respective treated groups (Supplemental Image 1B). This was also suggested by the more homogeneous clustering of samples and genes visible on the heatmap, with 2,000 genes exhibiting higher variance among control samples (Supplemental Image 1C) compared with the one that contained all analyzed samples (Supplemental Image 1D). Clustering observed on the heatmap with the 2,000 genes showing higher variance among all analyzed samples (Supplemental Image 1D) also suggested that, apart from effects on the immune system and negative regulation of cellular processes on day 20, passage of time appeared to have a higher global impact on CL expression of these genes than COX2 inhibition. This was further visualized using a principal component analysis (PCA) plot of the same 2,000 genes with higher variance among all samples (Figure 1). Thus, samples appeared to be segregated based on time-point of analysis, with those samples from days 5 and 10 being proximally distributed on a cluster and samples from days 20 and 30 on the other side of the plot. The scattered distribution of samples treated up to day 20 after ovulation indicates stronger effects of treatment in this group (Figure 1).

### Time-Dependent Effects

**Figure 2:**
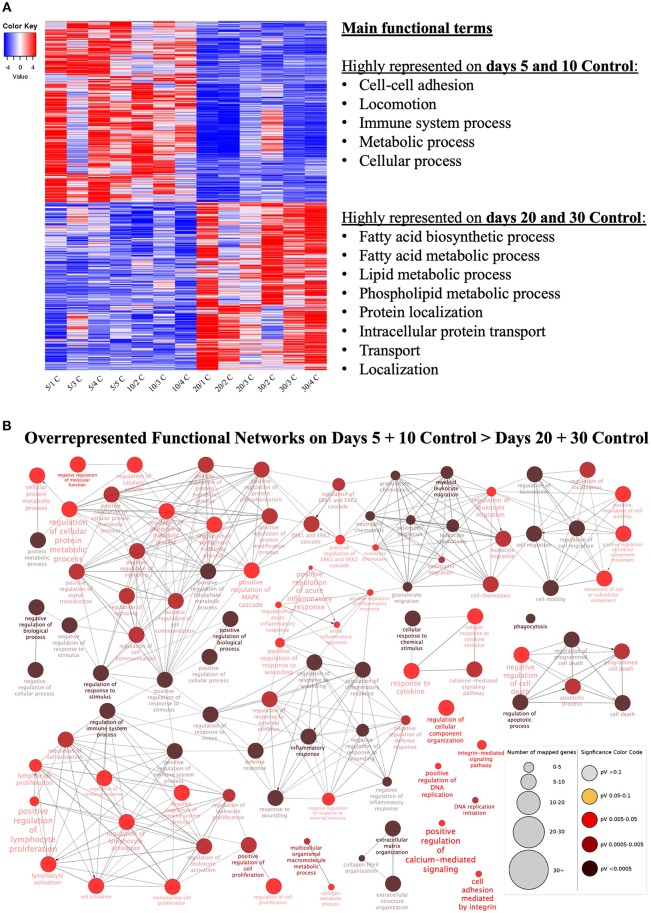
Functional categorization of differentially expressed genes (DEGs) affected at selected time points (contrast “days 20+30 control over days 5+10 control”). **(A)** Heatmap of 3,484 DEGs in the contrast “days 20+30 over days 5+10 control.” Gradient of high to low expression of each gene relative to average expression is indicated by red to blue colors. 1,803 genes were more highly expressed in early (days 5 and 10) CL stages whereas 1,681 genes were more highly expressed in mid-diestrus (days 20 and 30) stages (*P* ≤ 0.01, FDR ≤ 0.1). Representative overrepresented functional terms in each group of upregulated genes are listed (statistical details are provided in the text and Supplemental Table 3). Entire list of DEGs is provided as Supplemental Table 1. **(B)** Functional networks found for downregulated DEGs from the contrast “days 20+30 control over days 5+10 control.” Overrepresented functional terms of days “5+10 control” are shown. Redundant or non-informative terms were removed and the networks obtained were manually rearranged. Number of mapped genes is indicated by the node size, while significance of functional terms is denoted by node color (represented in legend at the right bottom corner). Networks more highly represented on days 5 and 10 control (developing gonadotropin-independent CL) were related to immune function, extra-cellular matrix (ECM), intracellular signaling and apoptosis.

Samples available for the present study were representative of different regulatory stages of early CL development, i.e., the early developing, gonadotropin-independent CL (days 5 and 10), and the mature CL in transition and during its dependence on gonadotropins (days 20 and 30, respectively). Close similarity between samples from days 5 and 10 on the one end, and days 20 and 30 on the other end, was also suggested from the PCA plot (Figure 1). Therefore, when evaluating the effects of time on the CL transcriptome, samples were grouped accordingly with differential expression analysis (pairwise comparison) being performed for the contrast “Days 20+30 control over days 5+10 control.” This analysis was performed using the DESeq2 package for Bioconductor and genes were assumed to be differentially expressed if *P* < 0.01 and FDR < 0.1. Of the total set of 19,856 genes, 13,332 genes were considered expressed (above the threshold of 10 reads per gene). DEGs accounted for 3,484 features with 1,681 being up- and 1,803 down-regulated at days 20+30 compared with the early developing CL (days 5+10; Table 2, Figure 2A). A detailed list of all DEGs affected by time is provided in Supplemental Table 1. Additional representation of DEGs in Volcano plots, filtered by FDR < 0.1 and log2Ratio > 0.5, is provided in Supplemental Image 2A. Following this, functional characterization of DEGs was performed. First, the pairwise comparison was classified according to Gene Ontology (GO) terms related to the domain biological process (BP). Panther software was used to calculate enrichment scores for each term, and results were further corroborated with Enrichr. Lists of up to ten (n = 10) representative genes for different functional terms, as well as upstream regulators and networks identified in the following steps, are presented in Supplemental Table 3.

**Table 2:** Summary of differential expression analysis (pairwise comparison) for all selected contrasts investigated in the present study.

**Contrasts**	**Days 20+30 C over days 5+10 C**	**Day 5 T over** **day 5 C**	**Day 10 T over day 10 C**	**Day 20 T over day 20 C**	**Day 30 T over day 30 C**
Total DEGs (*P* < 0.01, FDR < 0.1)	3,484	74	2	1,741	552
Number of genes with counts above threshold (10 reads per gene)	13,332	13,428	13,280	13,477	13,187
Upregulated genes	1,681	47	1	1,146	306
Downregulated genes	1,803	27	1	595	246

Genes more highly represented at early CL stages (days 5+10) compared to days 20+30 were strongly associated with (Figure 2A, Supplemental Table 3): cell-cell adhesion (*P* = 2.40E-3), locomotion (*P* = 1.28E-3), immune system process (*P* = 1.27E-3), metabolic process (*P* = 9.67E-4) and cellular process (*P* = 2.73E-4). On the other hand, functional terms over-represented in the mature CL (days 20+30) included (Figure 2A, Supplemental Table 3): fatty acid biosynthetic process (*P* = 1.26E-3), fatty acid metabolic process (*P* = 2.96E-4), lipid metabolic process (*P* = 1.12E-6), phospholipid metabolic process (*P* = 2.35E-3), protein localization (*P* = 1.64E-3), intracellular protein transport (*P* = 1.04E-3), transport (*P* = 3.12E-5) and localization (*P* = 1.22E-4).

Using as input the lists of upregulated and downregulated DEGs for this contrast (Supplemental Table 1), enriched functional networks were grouped and visualized with the ClueGO plug-in for the platform Cytoscape (Figure 2B, Supplemental Table 3, Supplemental Image 3A). Among the more highly represented functional networks observed on days 5 and 10 were those referring to cell signaling and metabolism, extracellular matrix, apoptosis and, in greater numbers, networks related to immune function (Figure 2B, Supplemental Table 3). On the other hand, networks mainly observed on days 20 and 30 were associated with intracellular transport and (lipids) metabolism (Supplemental Table 3, Supplemental Image 3A).

The prediction of the most significantly affected signaling pathways was obtained from IPA software by using the list of DEGs as input (*P* < 0.01, FDR < 0.1). Among the most enriched canonical pathways predicted to be activated by the passage of time were those related to (Supplemental Table 3): cholesterol synthesis/steroidogenesis (superpathway of cholesterol biosynthesis, *P* = 4.90E-5; cholesterol biosynthesis I, *P* = 1.20E-4; cholesterol biosynthesis II, *P* = 1.20E-4; and cholesterol biosynthesis III, *P* = 1.20E-4). On the other hand, among significant pathways that were predicted to be inhibited by time were those related to (Supplemental Table 3): cellular proliferation (EIF2 signaling, *P* = 7.94E-16; mTOR signaling, *P* = 3.98E-12), cell cycle (cyclins and cell cycle regulation, *P* = 3.63E-7), ECM production (inhibition of matrix metalloproteases, *P* = 1.86E-4) and immune function (leukocyte extravasation signaling, *P* = 3.55E-8; acute phase response signaling, *P* = 5.89E-6; IL8 signaling, *P* = 3.63E-5; chemokine signaling, 4.68E-5; dendritic cell maturation, *P* = 1.8E-4).

The analysis of DEGs with IPA allowed additional identification of upstream regulators possibly affecting expression of the DEGs obtained. Among the predicted upstream regulators, the following factors were identified: transforming growth factor β 1 (TGFβ1, *P* = 7.27E-33), tumor necrosis factor (TNF, *P* = 5.88E-26), beta-estradiol (*P* = 3.08E-22), platelet-derived growth factor BB (PDGF BB, *P* = 1.08E-18), interferon gamma (INFγ, *P* = 3.47E-15), progesterone (P4, *P* = 3.86E-15), insulin growth factor 1 (IGF1, *P* = 3.85E-13), nuclear factor kappa B inhibitor alpha (NFκBIα, *P* = 5.86E-13) and prostaglandin (PG) E2 receptor 2 (PTGER2/EP2, *P* = 9.5E-12) (Supplemental Table 3).

### Treatment-Induced Effects

#### Differential Expression Analysis (Pairwise Comparison) and Venn Diagram

The effects of treatment with Previcox on the transcriptome of CL tissue were assessed by differential expression analysis, in which treated samples were compared with control samples at the respective time-points (days 5, 10, 20 and 30). Thus, the following contrasts were defined: “day 5 treated over day 5 control,” “day 10 treated over day 10 control,” “day 20 treated over day 20 control,” and “day 30 treated over day 30 control.” As described for time-dependent effects, the DESeq2 package for Bioconductor was used to obtain lists of DEGs. The thresholds of *P* < 0.01 and FDR < 0.1 were applied to consider a gene as being differently expressed. For all contrasts, a total of 19,856 genes were identified; only genes with at least 10 reads were considered as expressed and included in further analyses. A summary of DEGs analysis is presented in Table 2, while full lists of DEGs in response to all treatments at every time-point are presented in Supplemental Table 2.

For the contrast at day 5, after being filtered (*P* < 0.01, FDR < 0.1), 74 genes were considered to be DEGs, of which 47 were upregulated and 27 downregulated after treatment. As for the contrast “day 10 treated over day 10 control,” the expression of genes varied greatly individually, and resulted in only 2 DEGs filtered by the applied *P*-value/FDR thresholds. Following this low number of DEGs found at day 10, no functional characterization could be performed for this contrast. In the contrast for day 20, 1,741 genes met the criteria of *P* < 0.01 and FDR < 0.1, making this the treatment-related contrast with the highest number of DEGs. Of these DEGs, 1,146 were more highly expressed in the day 20 treated group, while 595 were more highly expressed in the day 20 control group. Finally, for the contrast “day 30 treated over day 30 control” 552 genes were differently expressed (*P* < 0.01, FDR < 0.1), comprising 306 up- and 246 down-regulated features in response to prostaglandin withdrawal. Further representation of DEGs (FDR < 0.1; log2ratio > 0.5) for the contrasts “day 5 treated over day 5 control,” “day 20 treated over day 20 control,” and “day 30 treated over day 30 control” in form of Volcano Plots are shown in Supplemental Image 2B–D.

**Figure 3:**
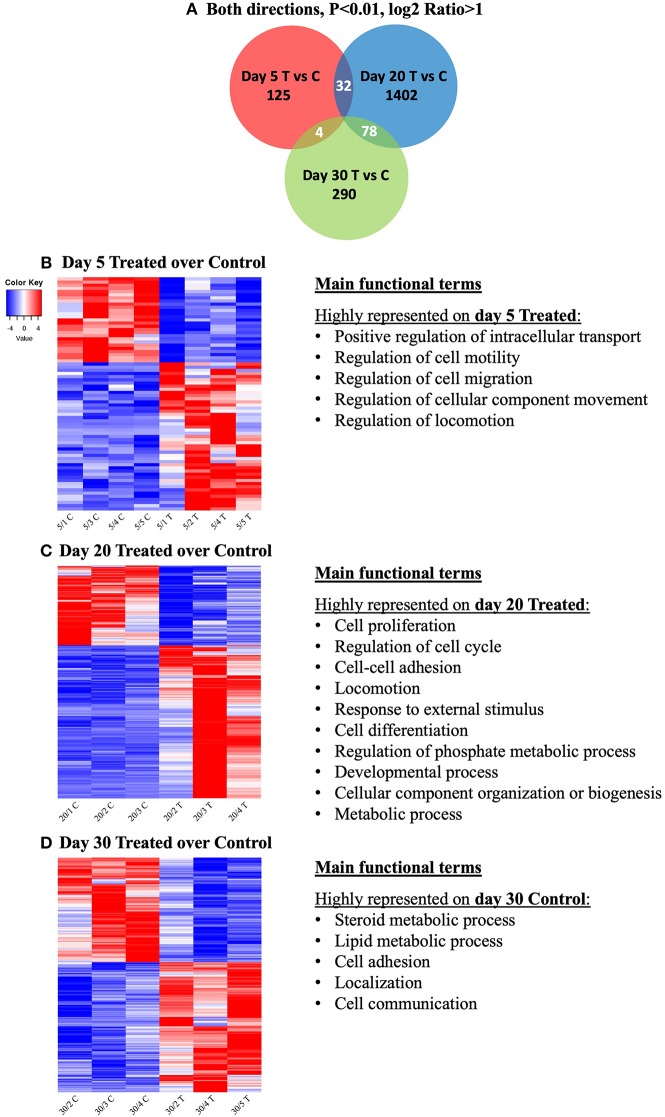
Venn diagram showing the distribution and overlap of differentially expressed genes (DEGs) induced by treatment at different time-points **(A)**. Lists of DEGs from the contrasts “day 5 treated over day 5 control,” “day 20 treated over day 20 control,” and “day 30 treated over day 30 control” were used as input data. Thresholds were defined for *P*-value < 0.01 and fold-change for up (log2Ratio ≥ 1) and downregulated (log2Ratio ≤ −1) genes. No gene was found to be concomitantly affected by treatment in all groups. Complete list of genes from each contrast and intersection are present in Supplemental Table 4. Heatmap and overrepresented functional terms present in differentially expressed genes (DEGs) induced by treatment at different studied time-points **(B–D)**. Gradient of high to low expression of each gene relative to average expression is indicated by red to blue colors. Main significant overrepresented functional terms (gene ontologies) in each group of upregulated genes are listed (statistical details are provided in the text and Supplemental Table 3). Entire list of DEGs is provided as Supplemental Table 2. **(B)** Heatmap of 74 DEGs of the contrast “day 5 treated over day 5 control” and representative significant overrepresented functional terms in “day 5 treated” are shown. **(C)** Heatmap of 1,741 DEGs of the contrast “day 20 treated over day 20 control” and representative significant overrepresented functional terms at “day 20 treated”. **(D)** Heatmap of 552 DEGs of the contrast “day 30 treated over day 30 control” and representative overrepresented functional terms in “day 30 control”.

In an attempt to identify genes that would be simultaneously affected by COX2-inhibition at different time-points of the CL life span, the intersections of DEGs from the contrasts at days 5, 20, and 30 were visualized with a Venn diagram (Figure 3A). The input DEGs were filtered for *P* < 0.01 and log2Ratio < −1 (downregulated) and log2Ratio > 1 (upregulated), and a complete list of genes from each intersection is provided in Supplemental Table 4. Thirty-two genes were simultaneously affected by treatment on days 5 and 20, while 78 were shared among days 20 and 30. Despite the time difference, 4 genes were still found to be simultaneously affected on days 5 and 30. However, no gene was concomitantly affected by prostaglandin withdrawal at the different time-points.

#### Functional Annotations, Networks, and Pathways

Further characterization of DEGs for each contrast was performed by identifying different enriched functional terms. The analysis flow was similar to the one applied in differential expression analysis of time-related effects: functional terms (GOs) were identified with Panther and Enrichr, functional networks were visualized with Cytoscape, and prediction of affected canonical pathways and upstream regulators involved was done by IPA. For input, DEGs were filtered by *P* < 0.01 and FDR < 0.1; lists of representative genes are provided in Supplemental Table 3.

##### Contrast “day 5 treated over day 5 control”

The main functional terms related to genes more highly expressed in treated samples from day 5 were (Figure 3B, Supplemental Table 3): positive regulation of intracellular transport (*P* = 3.48E-8), regulation of cell motility (*P* = 1.31E-6), regulation of cell migration (*P* = 7.40E-6), regulation of cellular component movement (*P* = 2.59E-6) and regulation of locomotion (*P* = 2.84E-6). Due to low numbers of input DEGs, no significant gene ontologies could be identified among the DEGs downregulated by treatment at this early gonadotropin-independent luteal phase. This also accounts for the Cytoscape analysis of functional networks from up- and down-regulated genes.

Enriched pathways predicted to be activated by IPA were mostly related to (Supplemental Table 3): cytoskeleton/cell movement/cell division (RhoA signaling, *P* = 1.10E-6; actin cytoskeleton signaling, *P* = 4.07E-5; signaling by Rho family GTPases, *P* = 6.31E-5; regulation of actin-based motility by Rho, *P* = 1.07E-4). Additionally, the pathways death receptor signaling (*P* = 5.50E-6) and leukocyte extravasation signaling (*P* = 2.75E-4) were also predicted to be activated by treatment, while the RhoGDI signaling (*P* = 5.13E-7) pathway was predicted to be deactivated. Among the predicted upstream regulators, actin alpha cardiac muscle 1 (ACTC1, *P* = 7.59E-6), PDGF BB (*P* = 3.13E-5), actin alpha 2 (ACTA2, *P* = 2.1E-4), TNF (*P* = 2.78E-4), and TGFβ1 (*P* = 9.94E-E4) were found (Supplemental Table 3).

##### Contrast “day 20 treated over day 20 control”

**Figure 4:**
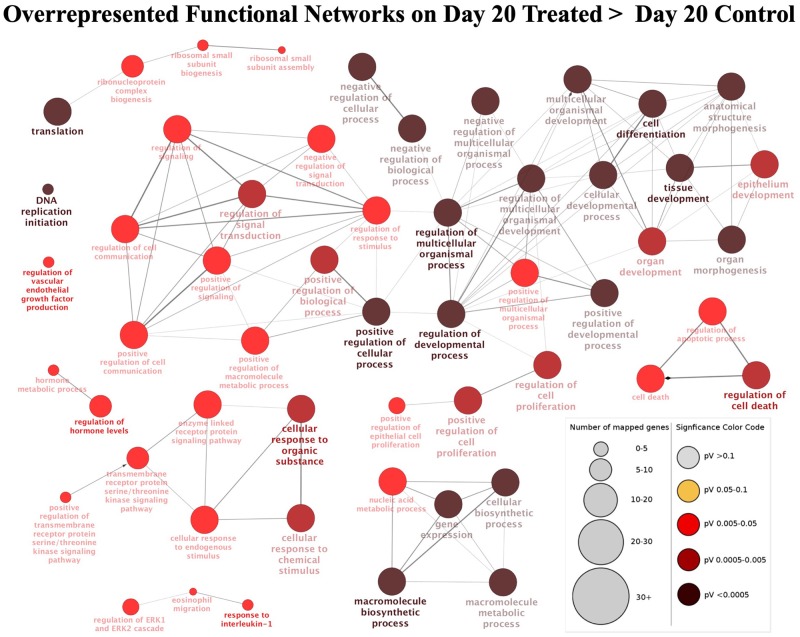
Functional networks found in upregulated differentially expressed genes (DEGs) from the contrast “day 20 treated over day 20 control.” Overrepresented functional terms of “day 20 treated” are shown. Redundant or non-informative terms were removed and the networks obtained were manually rearranged. Number of mapped genes is indicated by the node size while significance of functional terms is denoted by node color (represented in legend at the right bottom corner). Networks more highly represented in CL samples from animals treated until day 20 after ovulation were related to gene expression and translation, signaling, hormone regulation, cell differentiation and death, and immune function.

For this contrast, the following main terms more highly represented in the treated mature CL during its transition to gonadotropin dependence on day 20 were found (Figure 3C, Supplemental Table 3): cell proliferation (*P* = 4.78E-4), regulation of cell cycle (*P* = 7.06E-5), cell-cell adhesion (*P* = 9.85E-4), locomotion (*P* = 5.58E-5), response to external stimulus (*P* = 1.20E-3), cell differentiation (*P* = 1.84E-4), regulation of phosphate metabolic process (*P* = 2.03E-4), developmental process (*P* = 1.93E-5), cellular component organization or biogenesis (*P* = 6.12E-4) and metabolic process (*P* = 1.46E-3). High functional variation was found for DEGs in control samples on day 20, yielding only low enrichment scores without strongly enriched functional terms, and without any strongly enriched functional networks. This differed from the CL samples derived from dogs treated for 20 days with Previcox, in which strongly over-represented networks were found, referring to cell differentiation, cell death, gene expression, translation and signaling, hormone regulation and immune function (Figure 4, Supplemental Table 3).

In response to Previcox treatment, the significant canonical pathways that were predicted to be activated (Supplemental Table 3) were related by IPA analysis to cellular proliferation/growth (EIF2 signaling, *P* = 5.01E-21; mTOR signaling, *P* = 1.86E-9) and immune function (toll-like receptor signaling, *P* = 1.66E-4; adrenomedulin signaling pathway, *P* = 1.20E-3; and acute phase response signaling, *P* = 4.37E-3; NFκB signaling, *P* = 4.47E-3; IL6 signaling, *P* = 4.47E-3). Additionally, the relaxin signaling (*P* = 4.68E-4) pathway was also predicted to be activated while the angiopoietin signaling (*P* = 4.47E-3) pathway was predicted to be deactivated. The list of top upstream regulators for the observed effects included (Supplemental Table 3): β-estradiol (*P* = 6.66E-23), PDGF BB (*P* = 9.63E-20), TGFβ1 (*P* = 2.26E-15), IL1β (*P* = 1.1E-14), TNF (*P* = 4.27E-14), PGE2 (*P* = 3.68E-11), NFκBIA (*P* = 1.45E-10), Fas cell surface death receptor (FAS, *P* = 1.89E-9) and P4 (*P* = 2.14E-9).

##### Contrast “day 30 treated over day 30 control”

Genes higher expressed in day 30 control samples were related with the following functional terms (Figure 3D, Supplemental Table 3): steroid metabolic process (*P* = 2.32E-4), lipid metabolic process (*P* = 1.07E-4), cell adhesion (*P* = 1.33E-3), localization (*P* = 1.82E-3) and cell communication (*P* = 8.96E-4). No significantly enriched GO and networks could be found for genes upregulated in response to treatment on day 30, which was restricted by higher functional variation of identified DEGs. However, in control samples functional networks related to nitric oxide synthesis and angiogenesis were found to be highly enriched (Supplemental Table 3, Supplemental Image 3B).

Interestingly, canonical pathways predicted to be deactivated by treatment in mature CL at day 30 were related with (Supplemental Table 3): cholesterol synthesis/steroidogenesis (superpathway of cholesterol biosynthesis, *P* = 1.35E-7; cholesterol biosynthesis I, *P* = 6.31E-6; cholesterol biosynthesis II, *P* = 6.31E-6; cholesterol biosynthesis III, *P* = 6.31E-6), immune signaling (acute phase response signaling, *P* = 1.91E-4; NFκB signaling, *P* = 3.47E-4; TGFβ signaling, *P* = 5.13E-3; IL8 signaling, *P* = 6.61E-3; leukocyte extravasation signaling, *P* = 8.71E-3) and vascularization (VEGF signaling, *P* = 3.31E-3; PDGF signaling, *P* = 1.48E-3). With regard to cellular proliferation, the EIF2 signaling (*P* = 3.16E-13) pathway was predicted to be activated, while the mTOR signaling (*P* = 2.63E-7) pathway was predicted to be deactivated. Among the top upstream regulators predicted with IPA software were (Supplemental Table 3): TNF (*P* = 1.89E-10), peroxisome proliferator-activated receptor gamma (PPARγ, *P* = 9.22E-5), TGFβ1 (*P* = 9.58E-5), fibroblast growth factor 2 (FGF2, *P* = 1.44E-4) and NFκBIA (*P* = 2.25E-4).

### Expression of Candidate Target Genes

The expression of candidate genes was investigated by semi-quantitative (TaqMan) qPCR in 32 samples from the different available groups. All results for time-dependent and treatment effects are cumulatively presented in Supplemental Table 5. Extracted, significant results were prepared for the main document and are shown in Tables 3, 4. The functional groups chosen for validation of transcriptional analysis included: eicosanoid synthases (*TBXAS1, PTGDS*), immune factors (*TGF*β*1, TGF*β*R1, ICAM1, IDO1, NODAL, FAS, FASLG, NF*κ*B1, NF*κ*BIA*), growth factors (*PDGF B, FGF1, FGF2*), vascular regulators (*THBS1*), hypoxia-related factors (*EGLN1*/PHD2), nuclear receptors (*PPARG, NR4A1*) and steroid-related factors (*HSD17B7, SULT1E1*). Expression of all candidate genes was detected in all tissues and, generally, a good correlation was found between RNA-Seq and RT-qPCR results. Additionally, no significant changes in the expression of *EGLN1* and *PDGF B* could be found in response to treatment or passage of time.

**Table 3:** Relative gene expression of target candidate genes affected by time in control animals.

**Target**	**Group**	**RGE**	**SD+/SD–**	**ANOVA *P*-value**	**Dunn's test**
*TBXAS1*	Day 5 control	3.95	1.57/1.12	0.0006	5C vs. 20C – *P* < 0.05 10C vs. 20C – *P* < 0.01 10C vs. 30C – *P* < 0.05
	Day 10 control	6.02	8.98/3.6		
	Day 20 control	1.75	0.86/0.58		
	Day 30 control	2.25	2.66/1.22		
*PTGDS*	Day 5 control	6.17	6.69/3.21	0.0153	10C vs. 20C – *P* < 0.05
	Day 10 control	15.02	18.43/8.28		
	Day 20 control	4.39	3.95/2.08		
	Day 30 control	6.74	14.58/4.61		
*TGFβR1*	Day 5 control	6.28	3.51/2.25	0.0017	5C vs. 30C – *P* < 0.001 20C vs. 30C – *P* < 0.05
	Day 10 control	5.15	6.45/2.86		
	Day 20 control	6.49	4.86/2.78		
	Day 30 control	2.85	1.98/1.17		
*ICAM1*	Day 5 control	5.35	10.74/3.57	0.0003	5C vs. 20C – *P* < 0.01 5C vs. 30C – *P* < 0.01 10C vs. 20C – *P* < 0.05
	Day 10 control	3.23	1.76/1.14		
	Day 20 control	1.69	0.9/0.59		
	Day 30 control	1.83	0.77/0.54		
*FAS*	Day 5 control	2.61	1.01/0.73	0.0015	5C vs. 20C – *P* < 0.01 5C vs. 30C – *P* < 0.01
	Day 10 control	2.33	2.43/1.19		
	Day 20 control	1.4	0.38/0.3		
	Day 30 control	1.45	0.77/0.5		
*FASLG*	Day 5 control	4.59	2.66/1.68	<0.0001	5C vs. 20C – *P* < 0.01 5C vs. 30C – *P* < 0.001 10C vs. 20C – *P* < 0.05 10C vs. 30C – *P* < 0.01
	Day 10 control	4.2	2.05/1.38		
	Day 20 control	2.3	0.51/0.41		
	Day 30 control	1.85	1.13/0.7		
*NFκB1*	Day 5 control	1.63	0.63/0.46	0.0408	No significant effects in group comparisons
	Day 10 control	2.13	1.0/0.68		
	Day 20 control	1.35	0.72/0.47		
	Day 30 control	1.41	0.68/0.46		
*NFκBla*	Day 5 control	2.4	3.85/1.48	0.0006	10C vs. 20C – *P* < 0.001
	Day 10 control	4.11	6.81/2.56		
	Day 20 control	1.2	0.33/0.26		
	Day 30 control	1.75	0.88/0.58		
*FGF1*	Day 5 control	3.2	1.57/1.05	0.0031	5C vs. 30C – *P* < 0.01
	Day 10 control	5.47	8.77/3.37		
	Day 20 control	4.98	2.05/1.45		
	Day 30 control	6.45	3.23/2.15		
*FGF2*	Day 5 control	3.63	2.04/1.31	0.0032	20C vs. 30C – *P* < 0.01
	Day 10 control	3.45	2.18/1.34		
	Day 20 control	4.65	1.46/1.11		
	Day 30 control	2.22	0.82/0.6		
*THBS1*	Day 5 control	8.91	5.75/3.5	<0.0001	5C vs. 20C – *P* < 0.001 5C vs. 30C – *P* < 0.001
	Day 10 control	4.85	3.59/2.06		
	Day 20 control	1.97	1.4/0.82		
	Day 30 control	2.91	2.45/1.33		
*PPARγ*	Day 5 control	2.2	1.28/0.81	0.0183	10C vs. 20C – *P* < 0.05
	Day 10 control	4.08	6.75/2.54		
	Day 20 control	1.66	0.41/0.33		
	Day 30 control	1.89	0.9/0.61		
*HSD17B7*	Day 5 control	1.9	1.04/0.67	<0.0001	5C vs. 10C – *P* < 0.05 5C vs. 20C – *P* < 0.001 5C vs. 30C – *P* < 0.001
	Day 10 control	4.58	3.53/1.99		
	Day 20 control	7.7	1.74/1.42		
	Day 30 control	6.16	8.03/3.48		

*Relative gene expression (RGE) is presented for each group as the geometric mean and geometric standard deviation (SD). Non-parametric ANOVA (Kruskal-Wallis) analysis was followed by Dunn's test. Only results considered statistically significant (P < 0.05) are presented. Blue ANOVA indicates higher gene expression in early control groups (days 5 and/or 10); red ANOVA indicates higher gene expression in mid-diestrus control groups (days 20 and/or 30); black ANOVA indicates no specific changing pattern between early and mid-diestrus control groups (i.e., between days 5 and/or 10 and days 20 and/or 30)*.

**Table 4:** Relative gene expression of target candidate genes affected by treatment at each analyzed time-point.

**Target gene**	**TaqMan real time PCR**					**NGS results**	
	**RGE/contr**.	**SD+/SD–**	**RGE/treat**.	**SD+/SD–**	***P*-value**	***P*-value**	**Log2 ratio**
**A. Day 5 treated over control**							
*TGFβ1*	1.58	0.4/0.32	2.67	1.84/1.09	0.0017	0.02148	0.4632
*FASLG*	4.59	2.66/1.68	2.44	1.35/0.87	0.0013	0.03313	−1.4
*FGF2*	3.63	2.04/1.31	2.14	1.46/0.87	0.009	0.163	−1.136
*THBS1*	8.91	5.75/3.5	14.07	5.98/4.2	0.0129	0.2279	0.6146
*SULT1E1*	7.97	7.27/3.8	4.18	4.33/2.13	0.0023	0.003543	3.094
**B. Day 20 treated over control**							
*TBXAS1*	1.75	0.86/0.58	4.25	4.41/2.17	0.005	0.003262	2.014
*PTGDS*	4.39	3.95/2.08	28.31	28.06/14.09	<0.0001	7.43E-05	2.637
*TGFβR1*	6.49	4.86/2.78	2.18	1.91/1.02	0.0013	0.04251	−0.8656
*ICAM1*	1.69	0.9/0.59	16.76	23.31/9.75	<0.0001	4.21E-07	3.765
*NODAL*	4.5	11.64/3.24	262.07	227.7/121.8	<0.0001	2.98E-09	5.04
*FAS*	1.4	0.38/0.3	2.6	1.01/0.73	0.0003	0.006776	0.8808
*FASLG*	2.3	0.51/0.41	5.73	3.05/1.99	<0.0001	0.008149	2.836
*NFκB1*	1.35	0.72/0.47	2.42	1.04/0.73	0.0064	0.005073	0.932
*NFκBla*	1.2	0.33/0.26	5.69	2.62/1.8	<0.0001	7.41E-10	2.01
*FGF2*	4.65	1.46/1.11	2.37	2.41/1.19	0.0162	0.4982	−0.6664
*THBS1*	1.97	1.4/0.82	12.77	10.58/5.79	<0.0001	0.0007533	2.55
*PPARγ*	1.66	0.41/0.33	5.56	7.24/3.14	0.0006	7.94E-08	2.283
*NR4A1*	4.96	3.03/1.88	43.73	44.88/22.15	<0.0001	2.11E-07	3.616
*SULT1E1*	6.62	6.63/3.31	17.15	32.76/11.25	0.0396	0.001586	2.63
**C. Day 30 treated over control**							
*IDO1*	4.75	6.1/2.67	1.63	0.97/0.61	0.0003	0.0002207	−3.172
*FGF2*	2.22	0.82/0.6	3.57	2.36/1.42	0.0088	0.765	−0.2163

*Relative gene expression (RGE) is presented for each group (contr., control group; treat., treated group) as the geometric mean and geometric standard deviation (SD+/SD–). Student's t-test was applied to test the effect of treatment at each analyzed time-point. Only results considered statistically significant (P < 0.05) are presented. Blue t-test indicates higher gene expression in the control group, while red indicates higher gene expression in treated groups*.

#### Time-Dependent Effects

Changes in candidate gene expression associated with time were assessed in control samples. All significant effects observed with the passage of time (including statistical analysis) are presented in Table 3. Time-related effects were observed for: *TBXAS1*(*P* = 0.0006), *PTGDS* (*P* = 0.0153), *TGF*β*R1* (0.0017), *ICAM1* (*P* = 0.0003), *FAS* (*P* = 0.0015), *FASLG* (*P* < 0.0001), *NF*κ*BIA* (*P* = 0.0006), *FGF1* (0.0031), *FGF2* (*P* = 0.0032), *THBS1* (*P* < 0.0001), *PPAR*γ (*P* = 0.0183) and *HSD17B7* (*P* < 0.0001) (Table 3, Supplemental Table 5). Despite that *P* < 0.05 was obtained for *NF*κ*B* (*P* = 0.0408) with the ANOVA test, no significant effect was obtained in the multiple comparisons test (*P* > 0.05, Table 3, Supplemental Table 5).

The expression of most target genes was downregulated with the passage of time. Expression of *TBXAS1, ICAM1, FAS, FASLG*, and *THBS1* was significantly higher in early developing CL stages (days 5 and/or 10) and decreased toward CL maturation (days 20 and/or 30). The expression of *PTGDS, NF*κ*BIA*, and *PPAR*γ was significantly higher on day 10 than on day 20. *TGF*β*R1* and *FGF2* expression was the lowest on day 30 compared with days 5 and 20 or only with day 20, respectively. In a different direction, the expression of *FGF1* significantly increased from day 5 to day 30. Finally, *HSD17B7* had the lowest expression at day 5, increasing during later stages of CL development.

#### Treatment-Induced Effects

Effects of Previcox treatment were assessed in all available samples by comparing expression of candidate genes in treated and control groups at each time-point (days 5, 10, 20, and 30). No significant changes (*P* > 0.05) in the expression of *FGF1* and *HSD17B7* were obtained in response to treatment in any of the comparisons studied (Supplemental Table 5), even if their expression was predicted to be modulated by NGS on days 30 and 20. Additionally, no significant changes in the expression of any of the candidate genes could be observed on day 10 (Supplemental Table 5). In the pairwise comparison on day 5, *TGF*β*1* and *THBS1* exhibited increased expression in response to treatment, while *FASLG, FGF2* and *SULT1E1* were downregulated (for details, including statistical analysis, see Table 4A). At day 20, higher expression of *TGF*β*R1* and *FGF2* was observed in control samples (Table 4B). In a different direction, several factors were upregulated by treatment on day 20: *TBXAS1, PTGDS, ICAM1, NODAL, FAS, FASLG, NF*κ*B1, NF*κ*BIA, THBS1, PPARG, NR4A1*, and *SULT1E1* (Table 4B). Finally, on day 30, treatment decreased the expression of *IDO1* and increased the expression of *FGF2* (Table 4C).

## Discussion

### General Considerations

Among many species-specific regulatory features, the presence of a transitional gonadotropin independence in the developing canine CL is certainly one of the most intriguing (17, 20). It positions the dog as a valuable model for investigating CL development without the dominant effects of hypophyseal hormones that are observed, e.g., in livestock (42, 43). As described previously, PGs, mainly PGE2, are considered to be among the most important regulators of CL function during its independence from gonadotropins (3, 23, 24). It also became apparent that PGE2 might have a broader role in the canine CL, regulating luteal sensitivity to other hormones (e.g., PRL) and exerting vasoactive and immunomodulatory actions (3, 25, 26). Consequently, the wide spectrum of direct and indirect effects of PGs in the canine CL encouraged us to perform the present NGS-based study. The ultimate goal was to better understand the different roles that PGs might play in the CL transcriptome and, thereby, to elucidate other possible regulatory mechanisms induced by PGs in the CL. Taking advantage of access to control samples covering the time span between days 5 and 30 after ovulation, i.e., during the development and maturation of the CL, transcriptional changes were also assessed in these samples with regard to the effects of time. In agreement with our previous reports (3, 24, 26), large variations in gene expression were observed in the CL of Previcox-treated dogs. This may be at least partially explained by the small number of animals per group and individual variations in gene expression. However, as can be seen from the analysis of the sequencing data presented herein, treatment with Previcox itself seemed to be an additional cause for these fluctuations. In fact, samples from control groups showed a higher correlation with each other than samples from treated groups. This could also be observed on the heatmap analysis of the 2,000 genes with higher variance, showing more homogeneous clustering when only control samples were used. As discussed elsewhere, individual variations and pharmacokinetics may have played an important role in the lower homogeneity observed in treated groups (26). The evaluation of both PCA and heatmap plots also suggested a more homogeneous clustering of samples divided between early and mature CL than by application of treatment. It seemed, thus, that time had a great impact on the transcriptional changes observed among the samples studied.

### Time-Dependent Effects

Showing homogenous distribution of gene expression, with a clear distinction between the early and mature CL, time-dependent effects were examined in control samples and additionally served for quality control. When compared with developing CL (days 5 and 10), more highly represented genes in the mature CL (days 20 and 30) related to lipid biosynthesis/metabolism. The canonical pathways predicted to be upregulated at this stage were related to cholesterol biosynthesis. Cholesterol is a substrate required for steroid hormone synthesis. An increase of its production is probably required for the observed increased steroidogenic output from the CL at this time. As also confirmed by the qPCR analysis, the expression of *HSD17B7* (known as PRLR-associated protein) was found to increase with maturation of the CL. Together with other isoforms of 17βHSD, this enzyme is responsible for the conversion of estrone into estradiol, a more potent estrogen (44, 45). Considering the high variation in circulating 17β-estradiol (E2) during the canine diestrus (1), together with the concomitantly increased expression of aromatase (CYP19) (46), it is plausible that the observed increase in *HSD17B7* expression could be involved in the local provision of estrogens in the canine CL. Reflecting the increasing steroidogenic capacity of the CL, following their initial post-ovulatory decrease, E2 levels increase during the course of diestrus in the dog, roughly following the P4 secretion profiles (13, 47). The expression of the estrogen receptors, ESR1/ERα and ESR2/ERβ, has been confirmed in the canine CL (46). Nevertheless, the involvement of estrogens in regulating canine CL function remains underexplored, even though some attempts were made to shed light on the underlying mechanisms (6, 46). Functional terms more highly represented on days 5 and 10 after ovulation were mainly related to immune function and proliferative mechanisms, such as locomotion, cell-cell adhesion, extracellular matrix organization, and regulation of the ERK1/2 cascade. Accordingly, pathways related to cell cycle, proliferation and immune function, were predicted to be inactivated following CL maturation, i.e., at days 20/30 of the CL lifespan. The apparently increased immune activity in the developing canine CL is in accordance with its previously reported increased infiltration by macrophages, monocytes and lymphocytes at this stage (48, 49). Furthermore, regarding immune regulation, TNF was among the top predicted upstream regulators involved in the CL transcriptomic changes observed in response to time. Similarly, an increased expression of *TNF*α and its receptor *TNFR2* on day 5 compared with mature CL stages was reported previously (26). Here, the expression of *FAS* and *FASLG*, factors belonging to the TNF superfamily, was also found to be more highly expressed in early than in mature CL at mid-diestrus (days 5/10 over 20/30). Their increased expression at that time was corroborated by the qPCR results. The role of the FAS/FASLG system in luteolysis, as extrinsic inducers of apoptosis, has been widely described in different species (50–54). With regard to the early CL, FAS was also observed to be increased in bovine CL as early as at day 5 after ovulation, provoking the question of possible non-apoptotic FAS signaling in regulating CL function (55). Indeed, FAS has been shown to affect some downstream non-apoptotic signaling pathways, such as NFκB (56). Thus, although the role of FAS/FASLG in the developing CL is still unknown, it could be also related with immune-mediated tissue reorganization and proliferation. Similar to FAS/FASLG, thrombospondins (THBS) have been associated with luteolytic events, responding to PGF2α and acting as anti-angiogenic factors by inhibiting the pro-angiogenic FGF2 (57, 58). Similar to the rat (59), and as also found by our qPCR analysis, increased expression of *THBS1* was detected in early CL stages and decreased toward mid-diestrus in mature CL (days 20 and 30). Within the early CL stage, an intense angiogenic activity is observed. Thus, the increased expression of *THBS1* appears paradoxical and is not fully understood. It appears plausible that *THBS1* could act as a limiter of vascular overgrowth, as suggested by others (59). However, it should be mentioned that the protein availability of this factor, as well as the availability of its receptors, were not investigated in the present study, but certainly merit further attention.

Regarding eicosanoids, modulation of CL function is classically seen as a balance between the luteotropic function of PGE2 and the luteolytic activity of PGF2α. In the canine CL, the expression of PGE2 synthase (PTGES) and PGE2 receptor 2 (PTGER2/EP2) decreases with the passage of time (21). This expression pattern could explain inhibition of the eicosanoid signaling pathway predicted by IPA software. Accordingly, similar time-dependent effects were observed herein in the expression of *TBXAS1* and PGD2-synthase (*PTGDS)*, which decreased from the early to the mid-luteal phase in the transcriptional analysis (further confirmed by qPCR). Both eicosanoids have been predominanty characterized in other systems. Thus, whereas thromboxane 2 (TBXA2) has been related to platelet aggregation, myocardial ischemia and bronchoconstriction, PGD2 has been extensively described as a regulator of body temperature and sleep cycle, vasodilation, smooth muscles relaxation and bronchoconstriction (60–62). With regard to the reproductive systems, the inhibition of TBXAS leads to increased cAMP-dependent steroidogenesis in Leydig cells (63). As for PGD2, in males it acts as an activator of the Sox9 gene and is, therefore, pivotal in testicular organogenesis (64, 65). However, to the best of our knowledge, nothing is known about the modulatory effects these two eicosanoids might have on CL function. Strikingly, PGD2 can undergo spontaneous dehydration into different J prostanoids, such as 15d-PGJ2 (66, 67). This prostaglandin can mediate pro-inflammatory mechanisms through different pathways, but also affects anti-inflammatory responses, mainly through the nuclear receptor PPARγ (68). Expression of PPARγ decreased between days 10 and 20 in qPCR analysis, and is an alternative receptor for several different factors, including eicosanoids and fatty acids, with regulatory roles in fatty acid metabolism, cell differentiation and inflammation (69). Among other regulatory effects, PPARγ has an indirect role in potentiating the expression of STAR by upregulating cJUN (70). In the CL of pregnant dogs, it was stably expressed during the whole diestrus (39). However, here we observed increased expression of this receptor in early CL stages, similar to the two eicosanoid-synthases studied. Thus, it seems plausible that also in the dog PPARγ provides an alternative pathway for possible modulatory effects of PGD2-derived prostanoids in the CL.

Collectively, our analysis indicates that during the transition from the early developing to the mature CL a decrease in immune activity and tissue proliferation occurs, expectedly accompanied by its increased steroidogenic capacity. Additionally, among the predicted top upstream regulators, different hormones and PGE2 receptor 2 (PTGER2/EP2) were present, which are known to exhibit time-dependent changes in their expression in the CL during canine diestrus (14, 21). Taking into consideration that these functional changes and the expression of different genes were previously investigated and/or were expected, as mentioned elsewhere, the analysis of time-dependent effects served also as a primary validation of the Next Generation Sequencing methodology. Besides this, the expression patterns of *TBXAS, PTGDS, FAS/FASLG, NF*κ*B/NF*κ*BIA, THBS1, PPAR*γ, and *HSD17B7* in the canine CL were described and discussed for the first time herein. All of these factors may serve functional roles in the development of CL in the canine species and thus constitute topics worthy of more attention in the future.

### Treatment-Induced Effects

The functional suppression of COX2, and the consequent withdrawal of PGs, had variable effects on the different groups studied. The numbers of DEGs found for each studied time-point were also variable, being lower in gonadotropin-independent CL stages (days 5 and 10) than during the transition period and at gonadotropin dependency (days 20 and 30, respectively). Interestingly, the highest number of DEGs (1,741) was found in CL from dogs treated with Previcox for 20 days. This, together with the absence of genes commonly affected by treatment in all groups, reinforces the time and developmental stage-dependent effects of COX2 suppression on CL transcriptional activity. Furthermore, the presence of possible compensatory mechanisms for the withdrawal of PGs was suggested previously (3, 26) and appears to be a part of the inherent regulation of CL function in the dog. At the same time, the possible presence of such mechanisms advocates caution in the evaluation and interpretation of the results obtained because they might be induced by these mechanisms, rather than being directly linked to the function of PGs.

#### Gonadotropin-Independent Stage of CL

This stage relates to the early, developing CL treated with Previcox over 5 and 10 days. Large individual and functional variations in the response to treatment were observed at this time at the transcriptome level, with fewer genes passing the applied stringent *P*-value and FDR criteria. Nevertheless, functional terms related to cellular movement and division dominated at day 5 after ovulation in response to Previcox. Additionally, the predicted activation of cell movement and cytoskeleton-related pathways was mainly due to the increased expression of factors like actins, laminin and myosin observed in the transcriptome analysis. In preceding studies, significant effects of treatment at this time-point showed decreased expression of STAR, PRLR and PTGES, the latter one being associated with lower levels of intra-CL PGE2 (3). However, no significant effects were observed regarding transcriptional capacity, vascularization or immune function (24, 26). Here, as detected by qPCR, expression of the growth factor *FGF2* was decreased by Previcox treatment, while *THBS1* was upregulated. Considering the aforementioned interaction between these factors, the expression pattern of *FGF2* and *THBS1* suggests a disruptive effect of this treatment on angiogenic mechanisms. The inhibitory effects on vascularization appear even more plausible when the increased expression of *TGF*β*1* after treatment is considered. In fact, growth and capillary morphogenesis of endothelial cells isolated from bovine CL were diminished by this cytokine (71). Additionally, in the present study qPCR also detected decreased expression of *FASLG* in Previcox-treated CL. If, as described above, this factor is usually associated with anti-angiogenic activity, its increased expression in early CL stages could imply a positive role of *FASLG* in CL angiogenesis, which appears to be affected after treatment. Although hypothetical, this idea deserves further attention in the future.

Regarding other functional mechanisms, as indicated by qPCR, the gene expression of *SULT1E1* decreased after Previcox treatment. As indicated elsewhere, the auto/paracrine effects of estrogens in the canine CL have been proposed before (6, 46). SULT1E1 sulfoconjugates estrogens, disrupting their capacity to bind to their receptors and, in this way, preventing their actions on target tissues (72). Thus, the observed decrease of *SULT1E1* expression in response to treatment might disturb the balance of locally active estrogens in the CL, presumably as a part of the compensatory mechanisms following the withdrawal of PGs.

The effects of Previcox treatment on day 10 appeared less pronounced at the transcriptome level. In previous studies, however, we observed that inhibition of COX2 at day 10 decreased the expression of PTGES, consequently significantly decreasing the intra-CL levels of PGE2, and this was associated with a significant decrease in circulating P4 levels (3, 24). These previous findings apparently contrast with the low number of DEGs observed in the present analysis. It appears, however, plausible that the variation among the treated samples could be the culprit for the negative output from this analysis, in particular with regard to the applied adjusted *P*-value (FDR), accounting for multiple testing but not for the biological diversity in the response to the applied anti-COX2 insult.

#### Transition Toward Gonadotropin Dependence

This stage of the CL relates to the transitional period of development toward its gonadotropin dependence, represented in our study by day 20 after ovulation (17, 18). In fact, day 20 was the time-point most affected by treatment. At this time, the CL appears to be more susceptible to insults targeting its functionality. The main functional terms overrepresented in Previcox*-*treated samples at this day were related to cellular proliferation and immune response. This fits well with the previously reported reduced size of the nuclei of steroidogenic cells and increased expression of some pro-inflammatory interleukins (e.g., IL1β or IL6) in response to Previcox at this day (24, 26). In the present NGS analysis, IL1β and IL6 were also found to be differentially expressed after treatment. The activation of several immune system-related canonical pathways was predicted by IPA software, and increased expression of other pro-inflammatory factors, e.g., *ICAM1, NODAL, FAS, FASLG*, and *NF*κ*B1*, was further confirmed by qPCR. This apparently increased reactivity of the immune system was accompanied by negative effects induced by the treatment at this time-point on the steroidogenic machinery, mirrored in the decreased expression of 3βHSD and STAR (3, 24). These findings suggest predominantly negative effects of treatment on CL function at this stage.

With regard to vascular function, in addition to the previously found increased expression of endothelin-1 (END1) and downregulation of angiopoietin 1 (ANGPT1) at day 20 of treatment (26), here, increased expression of the anti-angiogenic *THBS1* and decreased levels of *FGF2* were identified. With this, the postulated negative impact of PGs withdrawal on CL vascular activity was further substantiated.

Contrasting with the decreased expression of *SULT1E1* at day 5 of treatment, its expression was elevated during the transitional phase at day 20 in the CL of Previcox-treated dogs. SULT1E1 was also elevated in the CL in mid-pregnant dogs undergoing luteolysis after treatment with an antigestagen (27), suggesting that the local withdrawal of estrogens could be related with decreased CL activity. The apparently suppressed expression of *HSD17B7* (*P* = 0.0608) at day 20 of treatment further strengthens the idea of possible involvement of estrogens in CL function. On the other hand, we also observed strongly increased expression of the nuclear factor *NR4A1* (also referred to as Nur77) in response to Previcox. This expression pattern was further confirmed by qPCR. Several functions were previously described for this receptor. The expression of NR4A1 was increased in the CL of bitches, cows and rats in response to treatment with the luteolysin PGF2α (73–75). This receptor is also known to be an important regulator of inflammation [reviewed in (76)]. Thus, it is plausible that the increased expression of this nuclear receptor might be related to the increased inflammatory response observed at this CL stage to the Previcox insult. Nevertheless, its exact functions in the canine CL remain to be determined. Due to the versatile effects of NR4A1 in different organs and systems, its possible actions on the CL appear worthy of more attention in the future.

The increased expression of *TBXAS, PTGDS*, and *PPAR*γ observed in transcriptomic and qPCR analyses at day 20 after Previcox treatment could represent possible compensatory mechanisms, as suggested in previous reports (3, 26). As mentioned elsewhere, PPARγ acts as an alternative receptor for prostaglandins (68, 69). Besides its potential to upregulate the pro-steroidogenic cJUN (70), it was shown to repress the activity of NFκB (77, 78). Indeed, increased expression of cJUN was proposed previously to counteract the negative effects of Previcox (26).

Cumulatively, in accordance with our previous findings (26), the functional inhibition of COX2, besides suppressing intra-CL PGE2 content (3), led to activation of CL immune system-related factors and pathways. It also negatively affected vascularization of the CL during its functional transition to the gonadotropin-dependent stage. The regulatory effects upon PPARγ and NFκB, the reciprocal interactions between FGF and THBS or even possible modulatory effects on locally produced estrogens, could possibly be involved in the maintenance of CL tissue homeostasis at this time in a PG-dependent manner.

#### Gonadotropin-Dependent CL Stage

In this comparison, the stage of CL development refers to mid-diestrus, represented by day 30. During this time, the maintenance of CL function is primarily dependent on PRL (19, 20). Day 30 showed the second highest number of DEGs in response to Previcox treatment. Also, in the Venn diagram analysis, days 20 and 30 shared the highest number of simultaneously affected genes, even if signaling pathways represented by these genes indicated their different functional status.

At day 30, the fully mature CL exhibits high steroidogenic capacity. This was reflected in the functional terms overrepresented at day 30 in control samples compared with their Previcox-treated counterparts. These included, e.g., steroidogenic and lipid metabolic processes, which were less represented in the CL of treated dogs. The analysis of functional pathways corroborated these observations, revealing cholesterol synthesis and steroidogenesis among the prevalent pathways affected by the treatment. Interestingly, in this contrast, the effects of the treatment appeared diverse and affected genes with higher functional variations. On the other hand, as indicated above, contrasting with day 20 of treatment, the immune system-related functional pathways (e.g., NFκB- and TGFβ-signaling) appeared less represented at day 30 in the treated CL. Their importance was, however, underlined by placing their respective associated factors among the top upstream regulators. Thus, besides TNF and PPARγ, TGFβ1 and NFκBIA (NFκB-associated factor) were also identified by IPA software. By adding new information, these results also fit well with the previously reported increased presence of CD4-expressing macrophages infiltrating the CL in response to treatment with Previcox at day 30 of treatment (26). Activation of the immune system was further indicated in the data set presented here by the strong suppression of IDO1 expression at day 30 of treatment. Being a rate-limiting enzyme in tryptophan catabolism, IDO1 function is considered to be a checkpoint in the activation of leukocytes by exerting immunosuppressive actions (79). Interestingly, and as indicated above, despite the proximities in the development time, maturation stage and steroidogenic capacity of the CL between days 20 and 30 after ovulation, the effects evoked upon the immune system at these two time-points by Previcox appeared to diverge, further highlighting the time-dependent effects of PGs withdrawal in the canine CL.

### Final Remarks

As shown in this and previous studies, treatment of dogs with Previcox affects multiple CL components and functions (3, 24, 26). In accordance with these observations, broad effects of COX2 inhibition were observed in the deep RNA-Seq analysis performed herein. These effects were clearly stage-dependent. Day 20, marking the transitional period toward gonadotropin-dependence, was the most affected by COX2-inhibition, identifying this period as the most sensitive stage of CL development to functional PGs withdrawal. It appears that at this stage the intrinsic regulatory mechanisms become unstable, while the luteotropic effects of PRL may be affected by treatment (3). This could also affect the strong compensatory mechanisms present in earlier stages, rendering the mature CL less capable of stabilizing its transcriptome in response to the insult caused by Previcox treatment. Indeed, it appears that the early CL is more resistant to PGs withdrawal, suggesting that this organ is intrinsically regulated and bears strong compensatory mechanisms. With maturation of the CL, its transcriptome becomes more sensitive to COX2 inhibition.

Mechanisms related to cellular proliferation, immune system and vascularization are undoubtedly involved in the proper development of the CL. Accordingly, here, deeper insights have been provided into the regulatory mechanisms underlying CL development, identifying several factors and pathways that could play roles in this process. Some of these, such as THBS1 and FAS/FASLG, are known for their involvement in the termination of CL function, but their role in CL formation is still not well-understood. Additionally, the modulatory effects of estrogens and PPARγ in the canine CL are still obscure and may play important luteotropic roles. Finally, the increased expression of *TBXAS* and *PTGDS* in early CL stages supports the idea that other PGs, besides PGE2, may play an important role in regulating and supporting the canine CL.

Apparently, by investigating transcriptomic effects, and being based on gene expression patterns, the information presented herein is not definitive and further functional and protein expression-related studies are needed to support these findings and hypotheses. Nevertheless, our analyses with Previcox-treated dogs clearly reveal broader regulatory roles linked to PGs in CL function, besides the luteotropic support of steroidogenesis by PGE2 or the luteolytic signaling of PGF2α. With this, the translational aspect of the present study in relation to other domestic animal species is obvious.

Our study falls into the clinical trials category and Previcox was used orally, as recommended by the manufacturer. This might have weakened effects on the target tissue due to its metabolism, even though 10 mg/kg of firocoxib, double the clinically recommended dosage, was used (in consultation with the manufacturer regarding safety). Despite causing disturbances in CL function, in none of the dogs was the luteal phase terminated. From the clinical point of view, this is important information because administration of Previcox appears to be safe for CL function and maintenance in non-pregnant, and presumably, also in pregnant dogs.

## Data Availability Statement

The datasets generated for this study can be found in NCBI's Gene Expression Omnibus, Series accession number GSE130369.

## Ethics Statement

Animal experiments were approved by the responsible ethics committee (permit 54/2008) of the University of Warmia and Mazury in Olsztyn, Poland.

## Author Contributions

MTP was involved in developing the concept of the present study, experimental design, generating data, analysis and interpretation of data, and drafting of the manuscript. FG was involved in knowledge transfer, and in the laboratory part of the project, as well as in critical discussion of data. HR contributed knowledge transfer, critical discussion and interpretation of data, and editing the manuscript. TJ was involved in design of the *in vivo* study and tissue collection, knowledge transfer, critical discussion of data, and revision of the manuscript. BH was involved in design of the *in vivo* study. BH and AB were involved in knowledge transfer, critical discussion of data, and revision of the manuscript. MPK designed and supervised the project, and was involved in interpretation of the data, and drafting and revision of the manuscript. All authors read and approved the final manuscript.

### Conflict of Interest

The authors declare that the research was conducted in the absence of any commercial or financial relationships that could be construed as a potential conflict of interest.

## Supplementary Material

Supplemental Table 1 List of differentially expressed genes (DEGs) found in the contrast “days 20+30 control over days 5+10 control.” All genes listed passed the criteria of *P* < 0.01 and FDR < 0.1 (False Discovery Rate, adjusted *P*-value).

Supplemental Table 2 List of differentially expressed genes (DEGs) found in the contrasts “day 5 treated over day 5 control,” “day 10 treated over day 10 control,” “day 20 treated over day 20 control,” and “day 30 treated over day 30 control.” All genes listed passed the criteria of *P* < 0.01 and FDR < 0.1 (False Discovery Rate, adjusted *P*-value).

Supplemental Table 3 Lists of representative genes (up to 10) and statistical analysis details of particular GO terms, particular functional networks, representative canonical pathways and top upstream regulators.

Supplemental Table 4 Complete list of genes presented in Venn diagram from the contrasts “day 5 treated over day 5 control,” “day 20 treated over day 20 control,” and “day 30 treated over day 30 control” and each intersection.

Supplemental Table 5 Relative gene expression and statistical analysis of all target candidate genes investigated with regard to time-dependent and treatment-induced effects. Relative gene expression (RGE) is presented for each group as the geometric mean and geometric standard deviation. For time-dependent effects, non-parametric ANOVA (Kruskal-Wallis) analysis was used, followed by Dunn's test. For the assessment of effects of treatment, Student's *t*-test was applied at each time-point analyzed. *P* < 0.05 was considered significant.

Supplemental Image 1 Initial explorative analysis of the sequencing dataset. Samples correlation matrix and heatmaps were obtained by using CountQC app provided in the SUSHI framework. In **(A,B)**, “K” and “Ctrl” relate to control samples while “P” and “Treat” relate to Previcox-treated samples. **(A)** Sample correlation matrix containing all samples submitted for RNA-Seq and considering all genes present. Samples 10/1 (10_K_1) and 30/1 (30_K_1) controls, and 10/2 (10_P_2) and 30/1 (30_P_1) treated, exhibited low correlation coefficients compared with other samples from the same group and were removed from further analysis. **(B)** Sample correlation matrix containing final list of samples used in the present analysis and considering all genes present. Control groups appear to have higher homogeneity than respective treated groups. **(C)** Heatmap of 2,000 genes with higher variance among all control samples. Gene ontologies (GOs) shown were obtained with Enrichr. Samples show apparent better clustering than in **(D)**, the heatmap of 2,000 genes with higher variance among all samples analyzed (control and treated).

Supplemental Image 2 Volcano plots of differentially expressed genes (DEGs; FDR < 0.1, Log2Ratio > 0.5) affected by the passage of time (**A**: contrast “days 20+30 control over days 5+10 control”) or induced by treatment (**B**: contrast “day 5 treated over day 5 control;” **C**: contrast “day 5 treated over day 5 control;” **D**: contrast “day 5 treated over day 5 control”).

Supplemental Image 3 Functional networks found in the upregulated differentially expressed genes (DEGs) from the contrast “days 20+30 control over days 5+10 control” and in the downregulated DEGs from the contrast “day 30 treated over day 30 control.” Functional networks were obtained with the ClueGO application for Cytoscape. The functional terms overrepresented in each group are shown. Redundant or non-informative terms were removed and the networks obtained were manually rearranged. Number of mapped genes is indicated by the node size while significance of functional terms is denoted by node color (represented in legend at the right bottom corner). **(A)** Networks more highly represented on days 20 and 30 control (mature CL) were related to intracellular transport and lipid metabolism. **(B)** Networks more highly represented in CL samples from control animals on day 30 after ovulation were related to nitric oxide synthesis and angiogenesis.
