# Supplementary figures and images for: Global Transcriptomic Analysis of the Canine corpus luteum (CL) During the First Half of Diestrus and Changes Induced by in vivo Inhibition of Prostaglandin Synthase 2 (PTGS2/COX2)
Source: Front Endocrinol (Lausanne). 2019 Nov 13;10:715. doi: 10.3389/fendo.2019.00715 (PMC6863809; doi:10.3389/fendo.2019.00715)

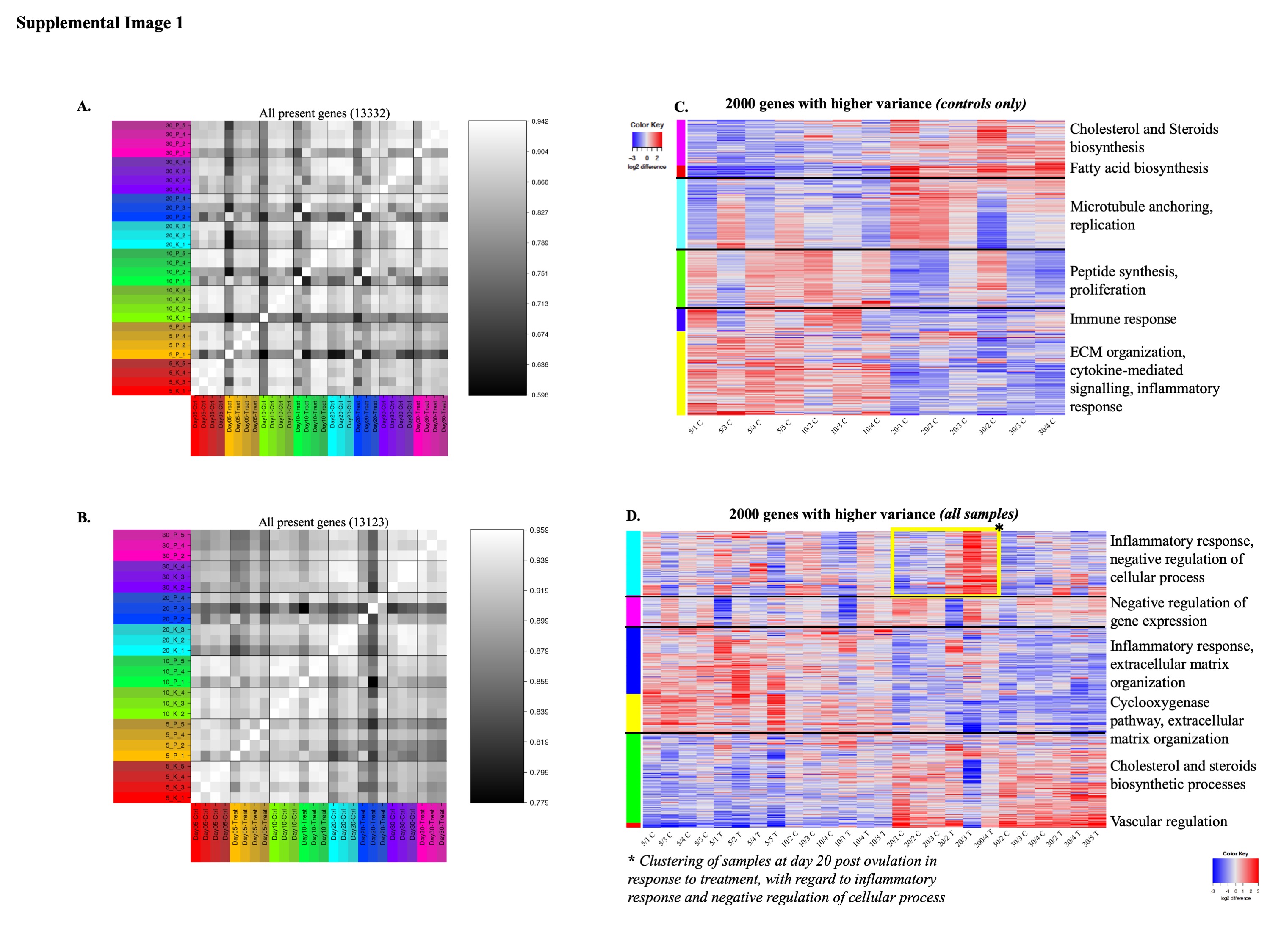

Supplement: Supplemental Image 1 — Initial explorative analysis of the sequencing dataset. Samples correlation matrix and heatmaps were obtained by using CountQC app provided in the SUSHI framework. In (A,B), “K” and “Ctrl” relate to control samples while “P” and “Treat” relate to Previcox-treated samples. (A) Sample correlation matrix containing all samples submitted for RNA-Seq and considering all genes present. Samples 10/1 (10_K_1) and 30/1 (30_K_1) controls, and 10/2 (10_P_2) and 30/1 (30_P_1) treated, exhibited low correlation coefficients compared with other samples from the same group and were removed from further analysis. (B) Sample correlation matrix containing final list of samples used in the present analysis and considering all genes present. Control groups appear to have higher homogeneity than respective treated groups. (C) Heatmap of 2,000 genes with higher variance among all control samples. Gene ontologies (GOs) shown were obtained with Enrichr. Samples show apparent better clustering than in (D), the heatmap of 2,000 genes with higher variance among all samples analyzed (control and treated). [file Image_1.jpg]

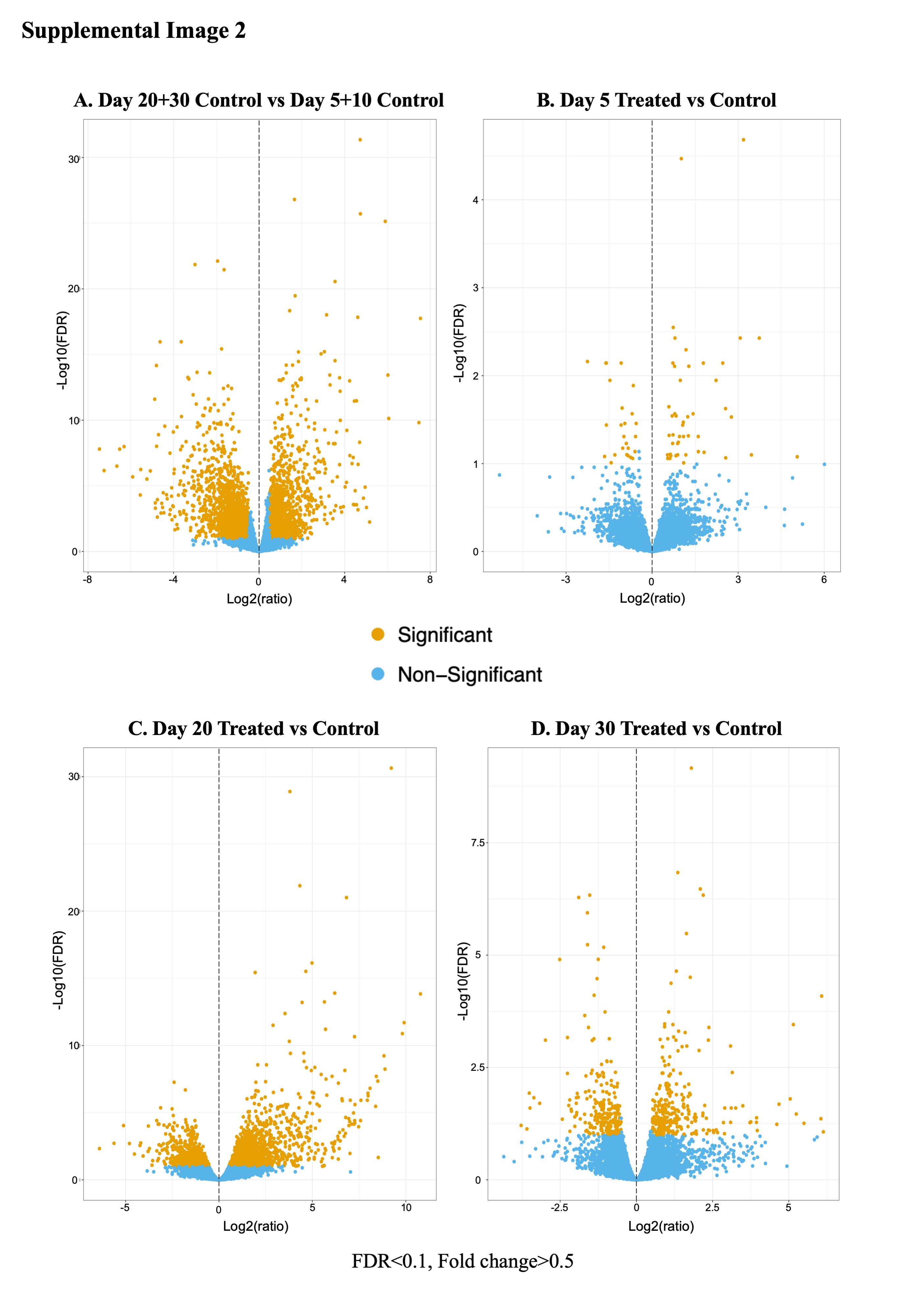

Supplement: Supplemental Image 2 — Volcano plots of differentially expressed genes (DEGs; FDR < 0.1, Log2Ratio > 0.5) affected by the passage of time (A: contrast “days 20+30 control over days 5+10 control”) or induced by treatment (B: contrast “day 5 treated over day 5 control;” C: contrast “day 5 treated over day 5 control;” D: contrast “day 5 treated over day 5 control”). [file Image_2.jpg]

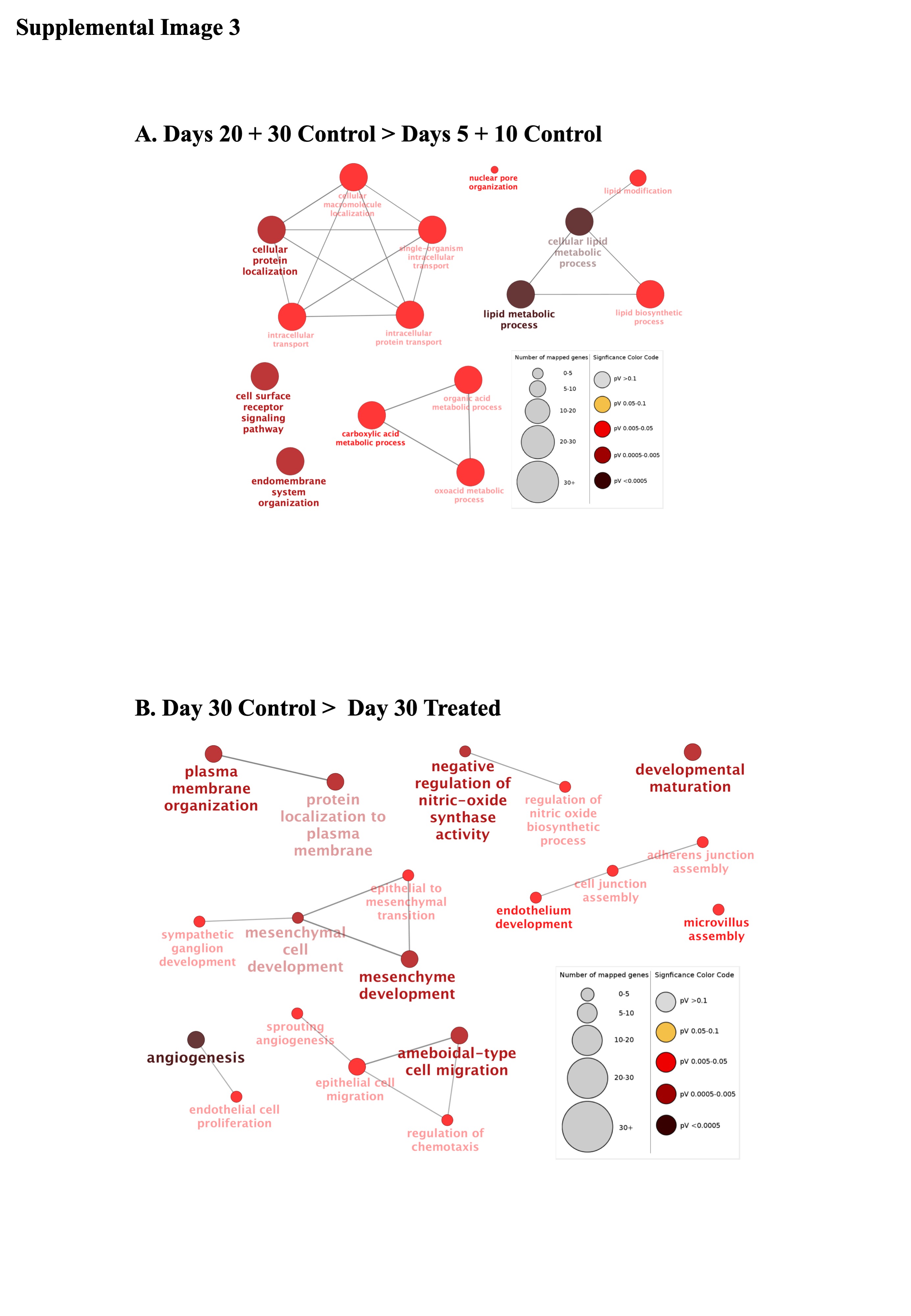

Supplement: Supplemental Image 3 — Functional networks found in the upregulated differentially expressed genes (DEGs) from the contrast “days 20+30 control over days 5+10 control” and in the downregulated DEGs from the contrast “day 30 treated over day 30 control.” Functional networks were obtained with the ClueGO application for Cytoscape. The functional terms overrepresented in each group are shown. Redundant or non-informative terms were removed and the networks obtained were manually rearranged. Number of mapped genes is indicated by the node size while significance of functional terms is denoted by node color (represented in legend at the right bottom corner). (A) Networks more highly represented on days 20 and 30 control (mature CL) were related to intracellular transport and lipid metabolism. (B) Networks more highly represented in CL samples from control animals on day 30 after ovulation were related to nitric oxide synthesis and angiogenesis. [file Image_3.jpg]
